# Pre-validation of a reporter gene assay for oxidative stress for the rapid screening of nanobiomaterials

**DOI:** 10.3389/ftox.2022.974429

**Published:** 2022-09-05

**Authors:** Sebastin Martin, Laura de Haan, Ignacio Miro Estruch, Kai Moritz Eder, Anne Marzi, Jürgen Schnekenburger, Magda Blosi, Anna Costa, Giulia Antonello, Enrico Bergamaschi, Chiara Riganti, David Beal, Marie Carrière, Olivier Taché, Gary Hutchison, Eva Malone, Lesley Young, Luisa Campagnolo, Fabio La Civita, Antonio Pietroiusti, Stéphanie Devineau, Armelle Baeza, Sonja Boland, Cai Zong, Gaku Ichihara, Bengt Fadeel, Hans Bouwmeester

**Affiliations:** ^1^ Division of Molecular Toxicology, Institute of Environmental Medicine, Karolinska Institutet, Stockholm, Sweden; ^2^ Division of Toxicology, Wageningen University and Research, Wageningen, Netherlands; ^3^ Biomedical Technology Center, Westfälische Wilhelms-University, Münster, Germany; ^4^ Institute of Science and Technology for Ceramics (ISTEC), CNR, Faenza, Italy; ^5^ Department of Chemistry, University of Torino, Torino, Italy; ^6^ Department of Public Health and Pediatrics, University of Torino, Torino, Italy; ^7^ Université Grenoble-Alpes, CEA, CNRS, IRIG, SyMMES, Grenoble, France; ^8^ Université Paris-Saclay, CEA, CNRS, NIMBE, Gif-sur-Yvette, France; ^9^ School of Applied Sciences, Edinburgh Napier University, Edinburgh, United Kingdom; ^10^ Université Paris Cité, CNRS, Unité de Biologie Fonctionnelle et Adaptative, F-75013 Paris, France; ^11^ Université Paris Cité, Unité de Biologie Fonctionnelle et Adaptative, Paris, France; ^12^ Department of Occupational and Environmental Health, Tokyo University of Science, Tokyo, Japan

**Keywords:** Nrf2, nanomaterial, interlaboratory validation, oxidative stress, nanotoxicology

## Abstract

Engineered nanomaterials have been found to induce oxidative stress. Cellular oxidative stress, in turn, can result in the induction of antioxidant and detoxification enzymes which are controlled by the nuclear erythroid 2-related factor 2 (NRF2) transcription factor. Here, we present the results of a pre-validation study which was conducted within the frame of BIORIMA (“biomaterial risk management”) an EU-funded research and innovation project. For this we used an NRF2 specific chemically activated luciferase expression reporter gene assay derived from the human U2OS osteosarcoma cell line to screen for the induction of the NRF2 mediated gene expression following exposure to biomedically relevant nanobiomaterials. Specifically, we investigated Fe_3_O_4_-PEG-PLGA nanomaterials while Ag and TiO_2_ “benchmark” nanomaterials from the Joint Research Center were used as reference materials. The viability of the cells was determined by using the Alamar blue assay. We performed an interlaboratory study involving seven different laboratories to assess the applicability of the NRF2 reporter gene assay for the screening of nanobiomaterials. The latter work was preceded by online tutorials to ensure that the procedures were harmonized across the different participating laboratories. Fe_3_O_4_-PEG-PLGA nanomaterials were found to induce very limited NRF2 mediated gene expression, whereas exposure to Ag nanomaterials induced NRF2 mediated gene expression. TiO_2_ nanomaterials did not induce NRF2 mediated gene expression. The variability in the results obtained by the participating laboratories was small with mean intra-laboratory standard deviation of 0.16 and mean inter laboratory standard deviation of 0.28 across all NRF2 reporter gene assay results. We conclude that the NRF2 reporter gene assay is a suitable assay for the screening of nanobiomaterial-induced oxidative stress responses.

## Introduction

It is a well-established paradigm that ambient particulate matter as well as engineered nanomaterials can trigger oxidative stress (Li et al., 2008; Stone et al., 2017). Under normal physiological conditions, reactive oxygens species (ROS) are continuously formed and immediately neutralized by antioxidant defences such as glutathione (GSH) and an array of antioxidant enzymes. However, under conditions of excessive ROS production, which may occur in cells exposed to engineered nanomaterials or other toxicants, the natural antioxidant defences of the cell may be overwhelmed (Sies and Jones, 2020). Oxidative stress is characterized by a cellular depletion of GSH while oxidized glutathione (GSSG) accumulates. Cells respond to this drop in the GSH/GSSG ratio by several protective or damage related signalling responses (Aguilano et al., 2014).

The NRF2–KEAP1 system plays a key role in maintaining redox homeostasis in eukaryotes (Sies and Jones, 2020). KEAP1 (Kelch-like ECH-associated protein 1) acts as a cysteine thiol-rich sensor of redox insults, whereas NRF2 (nuclear erythroid 2-related factor 2) is a transcription factor that regulates electrophile responsive element (EpRE)-mediated gene expression to switch on a battery of cytoprotective genes. Upon associating with other transcription factors, NRF2 binds to the EpRE and activates EpRE-mediated gene expression, including the genes encoding for detoxifying enzymes and proteins, such as glutathione peroxidase (GPx), NAD(P)H-quinone oxidoreductase (NQO1), superoxide dismutase (SOD), catalase (CAT), peroxiredoxin (PRx), glutathione S-transferase (GST), γ-glutamylcysteine synthetase (γ-GCS) and glutamate-cysteine ligase (GCL) genes. At higher levels of oxidative stress, this protective response is overtaken by cytotoxicity (Li et al., 2008).

Several recent *in vitro* studies reported the activation of the NRF2 pathway following exposure to a variety of engineered nanomaterials, including CeO_2_ nanomaterials (Choi et al., 2021), SiO_2_ nanomaterials (Cui et al., 2021), and ZnO nanomaterials (Zhang et al., 2021). Moreover, Kim et al. (2021) investigated seven different metal oxides (CuO, Co_3_O_4_, NiO, TiO_2_, CeO_2_, Fe_2_O_3_, and ZnO) using the ARE-NRF2 Luciferase KeratinoSens™ assay that is based on stably transfected immortalised human keratinocytes (HaCaT). CuO nanomaterials but not Co_3_O_4_, NiO, TiO_2_, CeO_2_, Fe_2_O_3_, or ZnO nanomaterials induced a positive response. The latter assay is recognized as a Test Guideline by the OECD since 2018 (Test No. 442D: *In vitro* Skin Sensitisation). Using a NRF2/ARE Responsive Luciferase Reporter HEK293 Cell Line, it has been shown that CuO, Mn_2_O_3_ and ZnO nanomaterials strongly induce the NRF2 mediated gene expression, while a recent study showed that Fe_2_O_3_ materials of different sizes induced limited gene expression in these reporter cells (Seleci et al., 2022). Using HEK293 cells, Ag nanomaterials have also been shown in several studies to trigger an NRF2 response in a range of different cell types (Miranda et al., 2022, and see other references therein).

Nanotoxicological studies have been conducted using a plethora of cell-based assays but there is a need for robust (validated) assays that are suitable for high-throughput screening of nanomaterials to improve safety assessment practices (Nymark et al., 2020). In the current study, we applied a reporter gene assay for the screening of oxidative stress induction by nanobiomaterials. This pre-validation study was performed within the EU-funded research project BIORIMA (“biomaterial risk management”). The overarching goal of the BIORIMA project has been to develop a risk management framework for nanobiomaterials used in medical devices and advanced therapy medicinal products (Giubilato et al., 2020). Hazard assessment of nanobiomaterials is one of the important elements of this framework and the approaches to assess the hazard potential of nanobiomaterials can either be based on methods adopted from classical toxicology (of chemicals and other particles) or on alternative methods, including *in vitro* and *in vivo* methods and *in silico* modelling (Giubilato et al., 2020).

In the present study, we pre-validated a reporter gene assay which is based on human osteoblastic osteosarcoma U2OS cells that express luciferase through transfection with a vector carrying antioxidant response elements (ARE) upstream of a luciferase reporter gene (van der Linden et al., 2014). Participating laboratories were recruited from the BIORIMA consortium, of which 7 laboratories fully completed all experiments. Cells were exposed to Fe_3_O_4_-PEG-PLGA (Fe_3_O_4_ -PolyEthylene Glycol - PolyLactide-co-Glycolide Acid) nanomaterials and to Ag and TiO_2_ “benchmark” nanomaterials. The Fe_3_O_4_-PEG-PLGA nanomaterials are envisioned for a variety of applications in medicine, including as a magnetic hyperthermia agent, an *in vivo* imaging/contrast agent, and an active targeting and drug delivery agent (Cazzagon et al., 2022), and for this reason, these materials were selected as one example of a relevant nanobiomaterial.

## Materials and methods

### Reagents

Curcumin (Sigma cat no. C1386), dichlorvos (Sigma cat no. 45441), and mannitol (Sigma cat no. M9647) were purchased from Sigma Aldrich (Amsterdam, Netherlands), and dimethyl sulfoxide (DMSO) (Arcos cat no. 167852500) was purchased from Acros Organics (Geel, Belgium). Dulbecco’s Modified Eagle Medium with Ham’s Nutrient Mixture F-12 (1:1) (DMEM/F12) without phenol red (Gibco cat no. 31331-028), Trypsin 0.5% EDTA (10x) (Gibco cat no. 15400-054), nonessential amino acids (NEAA) (Gibco cat no. 11140-035), and phosphate-buffered saline (Gibco cat no. 20012019) were from Gibco (Carlsbad, CA), geneticin (G418) (Duchefa cat no. G0175001) from Duchefa (Haarlem, Netherlands), and penicillin/streptomycin, pH 7.4 (P/S) (Invitrogen cat no. 15070063) from Invitrogen (Breda, Netherlands). Fetal bovine serum (FBS) (Gibco cat no. 10270-106) and dextran-coated charcoal-stripped fetal calf serum (DCC-FCS) (Gibco cat no. 12676029) were both purchased from Gibco.

### Nanobiomaterials

Fe_3_O_4_-PEG-PLGA nanomaterials were provided by Colorobbia Holding S.p.A (Firenze, Italy) in the framework of the BIORIMA research project and synthesized as described (D'Elios et al., 2018). Both Ag and TiO_2_ nanomaterials (designated NM300K and NM101, respectively) were from the nanomaterial repository of the Joint Research Center of the European Commission (Ispra, Italy). Ag nanomaterials were provided as a suspension. The NANOGENOTOX protocol was used for dispersion of TiO_2_ (Farcal et al., 2015).

### Characterization of particles

#### Small angle X-ray scattering

The dissolution and aggregation of the Ag nanomaterials was monitored by small angle X-ray scattering (SAXS) following incubation for 18 days at 37°C in MEM media (Invitrogen cat no. 51200) supplemented with 4% FBS (Sigma cat no. F7524), 1% Glutamax (Invitrogen cat. no. 35050-038), 1% non-essential amino acids (Invitrogen cat no. 11140), 1% sodium pyruvate (Sigma cat no. S8636), 1% penicillin-streptomycin (Invitrogen cat no. 15140-122) and 1% HEPES (Invitrogen cat no. 15630). SAXS measurements were carried out on Xeuss 2.0 (Xenocs) and ChemSaxs (lab design, CEA) high-resolution X-ray spectrometers in Kapton capillaries at a concentration of 0.5 mg/ml. The signal of the baselines was subtracted and data were fitted with PySAXS software (https://pypi.org/project/pySAXS/). SAXS experiments were performed by one of the participating laboratories.

#### Transmission electron microscopy

TEM was performed by using a FEI TECNAI F20 microscope operating at 200 keV. The suspension was drop-casted on a holey carbon film supported by a gold grid. The specimen was then dried at 60°C. To gather information about particles morphology the images were taken in phase contrast mode and high-angle annular dark-field scanning transmission mode (HAADF-STEM). High resolution (HREM) and Selected Area Electron Diffraction (SAED) analyses were performed to investigate the crystalline phase structure and composition. To calculate the mean particle diameter more than 100 particles were measured. TEM experiments were performed by one of the participating laboratories.

#### Dynamic light scattering

Hydrodynamic sizes and zeta potential of Fe_3_O_4_-PEG-PLGA nanomaterials were determined as previously described in the NanoREG project (Bhattacharya et al., 2017). In short, concentrations of the test samples were adjusted from the 1 mg/ml respective stock suspensions using either endotoxin free water or the medium with or without FBS to a concentration of 25 μg/ml for the measurements. Particle size distribution and zeta potential of the test samples were measured by dynamic light scattering (DLS) technique using Malvern Zetasizer Nano ZS. Three measurements with no pause were taken for particle size distribution and for the zeta potential values of each test material at 0, and 24 h at a temperature of 25°C. DLS experiments were performed by one of the participating laboratories.

### Endotoxin detection

The Limulus Amoebocyte Lysate (LAL) assay was applied to detect bacterial endotoxin contamination as described earlier (Kroll et al., 2013; Eder et al., 2022). The Limulus Amoebocyte Lysate PYROTELL^®^–T assay was purchased from Associates of Cape Cod, Inc. (East Falmouth, MA) and used according to the manufacturer’s instructions. Data analysis was performed using PYROS^®^ Software (Associates of Cape Cod, Inc.).

### Cell culture

U2OS-NRF2 cells were kindly provided by Bio Detection Systems (Amsterdam, Netherlands). The human osteoblastic osteosarcoma U2OS-NRF2 cells (van der Linden et al., 2014) express two oligos containing four different EPRE sequences: 1 × consensus EPRE (TCA​CAG​TGA​CTA​AGC​AAA​AT), 1 × hNQO1 EPRE (TCACAGTGAC TCAGCA-GAAT), 1 × hGCLM EPRE (AGA​CAA​TGA​CTA​AGC​AGA​AA) and 1 × hGCLC EPRE(TCACAGTCAGTAAGTGATGG). The two oligos were ligated into a promoter-less luciferase reporter-construct pLuc. Because the U2OS cells express the NRF2 pathway endogenously, a selection construct (pSG5-neo) was used. The cells were cultured in DMEM/F12 supplemented with 10% FCS and penicillin/streptomycin (final concentrations 10 U/ml and 10 μg/ml, respectively) (designated as growth medium). Once per week, 200 μg/ml G418 was added to the culture medium to maintain selection pressure. Cells were maintained at 37°C in a humidified atmosphere with 5% CO_2_.

### Cell viability assay

Cytotoxicity of nanobiomaterials was evaluated by the Alamar blue (resazurin) assay as described (Keshavan et al., 2021). The cell viability experiments were performed by one of the participating laboratories, prior the “round robin” pre-validation experiments. The cells were trypsinized, counted, and resuspended in cell culture medium without phenol red and supplemented with 5% dextran-coated charcoal-stripped FCS (DCC–FCS), to a final concentration of 10^4^cells/well (100 µl). Cells were seeded in 96-well plates and exposed to test materials or were maintained in DCC–FCS alone (negative control). The assay reagent (Thermo Scientific, Sweden) (10% [v/v] solution of AlamarBlue^®^ reagent) was added to each well to monitor the cellular metabolic function. The samples were analyzed using a spectrophotometer (Tecan Infinite^®^ F200).

### Reporter gene assay

The potential induction of NRF2 mediated gene expression by nanobiomaterials was tested by measuring the induction of luciferase activity in the NRF2-U2OS cells. Protocols are available upon request. In brief, the cells were trypsinized, counted, and resuspended in cell culture medium without phenol red and supplemented with 5% dextran-coated charcoal-stripped FCS (DCC–FCS) at a final concentration of 10^4^cells/well (100 µl) in a 96-well plate without using the most outer wells. The plates were incubated for 24 h in a humidified atmosphere at 37°C under 5% CO_2_. Following this pre-incubation one reference plate was exposed containing 9 serial dilutions in the range of 1 × 10^−4^ M to 1 × 10^−8^ M (log10 dilution steps) of the reference compound curcumin, as well as a positive control dichlorvos (1 × 10^−5^–7 × 10^−7^ M) and a negative control mannitol (1 × 10^−3^–1 × 10^−5^ M). Dichlorvos was included as a positive control as it is known to induce a response in this assay, while the negative control (i.e., mannitol) should not. Curcumin was chosen as reference compound, as it usually results in a dose-effect response in the current assay. It is good practice to select different chemicals as reference chemical and positive control. The cells were exposed to reference compounds by adding the compounds from a 200 x concentrated stock solution in DMSO to exposure medium (5% DCC-FCS in DMEM/F12 without phenol red). Following exposure to the test materials, cells were further processed for the luciferase induction assay. Cells were rinsed using PBS followed by lysis through 30 µl low salt buffer (Tris, 25 mM, DTT 2.0 mM, CDTA 2.0 mM), and a subsequent freezing step at −80°C ensured complete cell lysis. Luciferase was measured using a flash mix protocol (BDS, Amsterdam, Netherlands). The flash mix or illuminate mix contained 20 mM tricine, 1.07 mM (MgCO_3_)4 Mg(OH)2.5 H_2_O, 2.67 mM MgSO_4_ x 7 H_2_O, EDTA 0.1 mM, DTT 1.5 mM, D-Luciferine 539 mM, ATP 5.49 mM. The measurements were performed in the different laboratories using a luminometer with two injectors, one to initiate the reaction (through the addition of the Luciferin present in the illuminate mix) and one for stopping the enzymatic reaction with NaOH. The reaction was thus stopped by adding 100 µL of 0.2 M NaOH. A threshold of induction factor of 1.5 was set for the NRF2 mediated gene expression, as described before (van der Linden et al., 2014).

### Design of “round robin” pre-validation

Fe_3_O_4_-PEG-PLGA nanomaterials were selected as a representative and novel nanobiomaterial for the present study. These nanomaterials are envisioned both for therapeutic and diagnostic applications. The “benchmark” TiO_2_ nanomaterials were included as an inert (non-cytotoxic) nanomaterial and Ag nanomaterials were included as a nanomaterial that most likely would elicit NRF2 mediated gene expression (based on the available literature, see above), though cytotoxicity at higher concentrations of the latter nanomaterials could not be excluded. Additional positive and negative chemical controls (dichlorvos and mannitol) were included for the assay based on the manufacturer’s recommendations.

**FIGURE 1:**
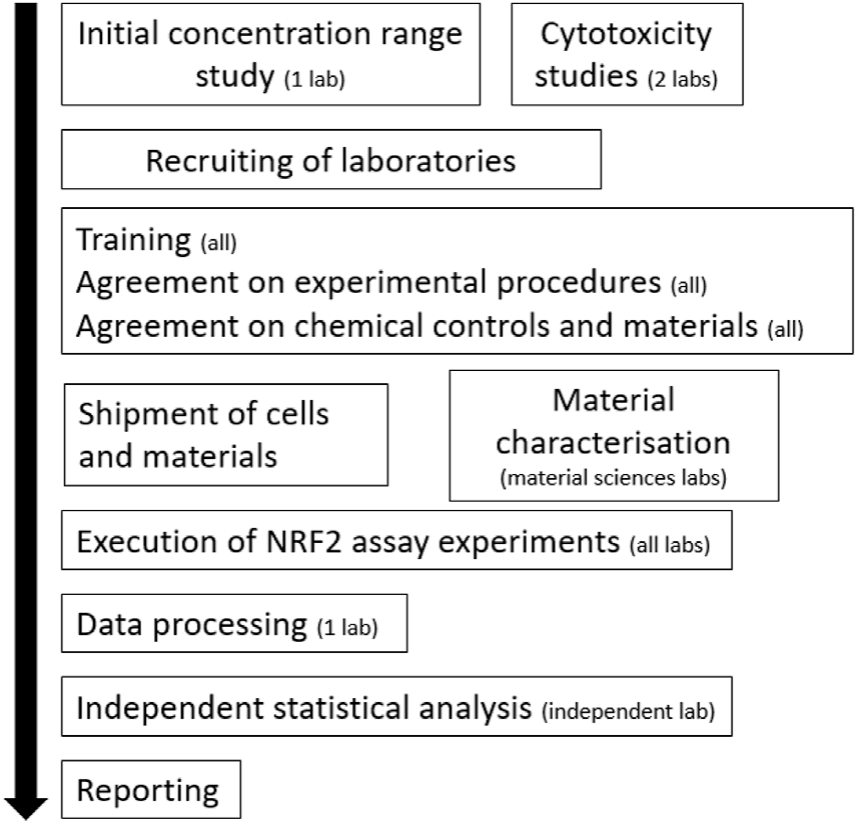
Workflow of the pre-validation experiments. The number of laboratories involved is indicated.

The participating laboratories were trained (online) on the execution of the NRF2 reporter gene assay, quality control measures, and data analysis (for a schematic of the workflow, refer to Figure 1). The following laboratories/institutions participated in the pre-validation study: Karolinska Institutet, Wageningen University, University of Torino, Université Grenoble-Alpes, Edinburgh Napier University, University of Rome Tor Vergata, Université Paris Cité, and Tokyo University of Science. However, one of these laboratories only tested Fe_3_O_4_-PEG-PLGA and not the other “benchmark” nanomaterials and the results are therefore shown separately. Protocols were extensively discussed and agreed upon during online meetings and tutorials. Chemicals and cell culture reagents were procured from the same source, and the NRF2-U2OS cell line was distributed to all the laboratories. The plate layout for the reporter gene assay was decided. Each experiment thus included one reference plate and three experimental plates. The three upper rows (B-C-D) of the reference plate as well as each experimental plate contained a full concentration range of the reference compound curcumin dissolved in DMSO. The lower part (rows E-F-G) contained the positive and negative control (reference plate) or one of the three nanobiomaterials under investigation (Fe_3_O_4_-PEG-PLGA, TiO_2_, Ag). The participating laboratories also harmonized the exposure conditions. Hence, Fe_3_O_4_-PEG-PLGA and Ag nanomaterials were diluted from a stock of 3000 μg/ml in dispersant provided with the particles at a concentration range of 0.21 µg/ml–3000 μg/ml followed by a second 30 x dilution step in exposure medium to an exposure range of 0.001–100 μg/ml. For TiO_2_, freshly prepared suspensions were made using the NANOGENOTOX dispersion protocol (Farcal et al., 2015). The reporter cells were exposed for 24 h in a humidified atmosphere at 37°C under 5% CO_2_.

### Data analysis

Data were exported to Excel (Microsoft) for further processing. Cytotoxicity was expressed as % viability towards the unexposed cells. For the NRF2 reporter gene experiments, the results were presented as the Induction Factor (IF), which is the measured relative light unit (RLU) value divided by the mean RLU value of the solvent control. When the induction factor of curcumin was over 8, the NRF2-U2OS reporter gene assay was regarded to be effective. Samples presenting 1.5 fold or higher induction were considered as inducers of NRF2 mediated-gene expression (van der Linden et al., 2014). Graphs were prepared in Prism 9.0 (GraphPad Software, Inc.) by analysing data using non-linear curve fitting (agonist versus response). To evaluate the variability of results and reproducibility of the assay, both the intra-laboratory and inter laboratory standard deviations were calculated across all NRF2 reporter gene assay results and plotted in a heatmap. Statistical analysis was performed using GraphPad Prism version 8.3.0.

Interlaboratory standard deviation of the assay results of all participating laboratories was calculated in accordance with ISO standards 5725-1 and 5725-2 for accuracy (trueness and precision) of measurement methods and results.

## Results

### Characterisation of nanobiomaterials

**FIGURE 2:**
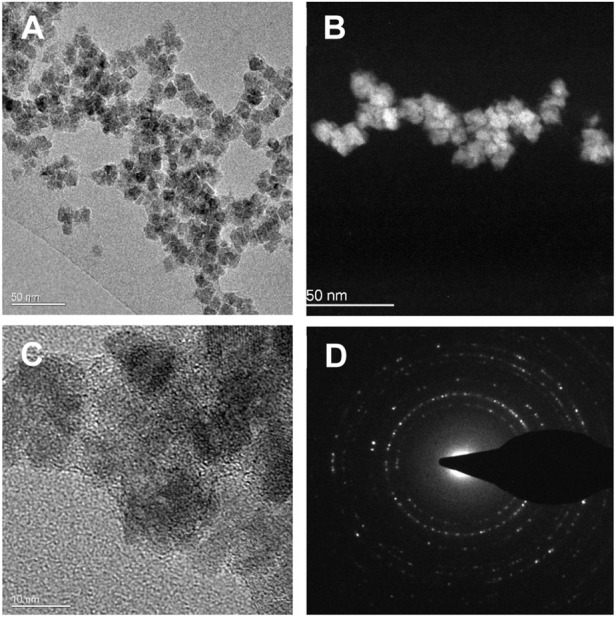
Transmission electron microscopy images of as-synthesized Fe_3_O_4_-PEG-PLGA nanomaterials. **(A)** TEM phase contrast image; **(B)** HAADF-STEM image; **(C)** HREM phase contrast image; **(D)** SAED polycrystalline pattern rings. Scale bars: **(A,B)** 50 nm; **(C)** 10 nm.

The Fe_3_O_4_-PEG-PLGA nanomaterials obtained from Colorobbia and the corresponding dispersant were evaluated for sterility (endotoxin content). Both were found to contain endotoxin levels below the US FDA-mandated level for medical devices (data not shown). The Fe_3_O_4_-PEG-PLGA nanomaterials were visualized by TEM. TEM phase contrast images (Figure 2A) and HAADF-STEM images (Figure 2B) indicated regular morphology with a mean particle diameter of 12 ± 4 nm. The higher magnification HREM phase contrast images (Figure 2C) disclosed a cubic crystal structure consistent with the magnetite lattice, and polycrystalline pattern rings collected by SAED (Figure 2D) were indexed as crystalline magnetite, identified as the unique phase composition. The benchmark materials were fully characterised, see Comero et al. (2011) for Ag, and Rasmussen et al. (2014) for the TiO_2_ nanomaterials.

SAXS analysis showed that there was little or no dissolution of the Ag nanomaterials following incubation at 37°C for 18 days in culture media. The average size of these nanomaterials did not change during incubation (15 ± 0.2 nm and 15 ± 0.2 nm at t = 0 and t = 18 days, respectively). The nanobiomaterials were also analysed with respect to hydrodynamic diameter and zeta potential in the relevant cell culture medium. Previously the dissolution of the Fe_3_O_4_-PEG-PLGA nanomaterials in cell culture media was shown to be less than 0.5% within 24 h (data not shown). Together the data indicated that all the test materials were stable following incubation in cell culture medium for 24 h at the exposure conditions for the NRF2 reporter gene assay (Supplementary Figures S1A,B).

### Cytotoxicity assessment

**FIGURE 3:**
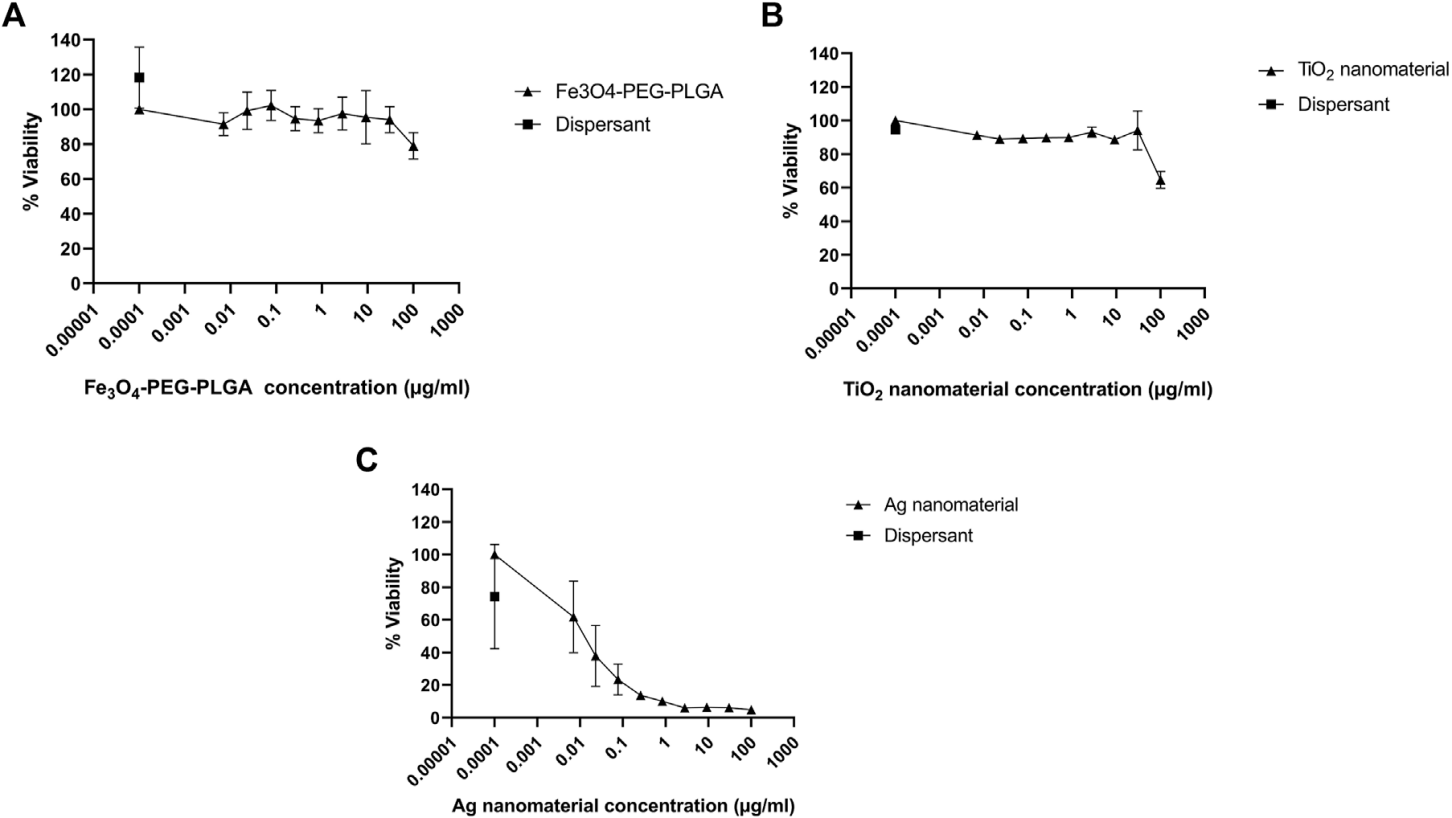
Cytotoxicity assessment. U2OS cells were exposed for 24 h to **(A)** Fe_3_O_4_-PEG-PLGA, **(B)** TiO_2_ nanomaterials, and **(C)** Ag nanomaterials or dispersants and cell viability (metabolic capacity) was evaluated using the Alamar blue assay. Data are mean values ± S.D. of three independent experiments.

**FIGURE 4:**
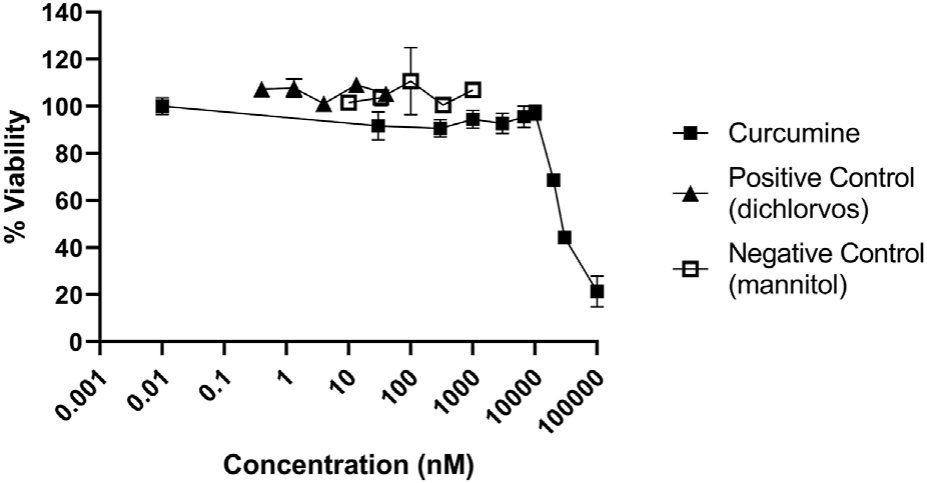
Cytotoxicity of control chemicals used in the NRF2 reporter gene assay. U2OS cells were exposed to curcumin, dichlorvos, and mannitol for 24 h and evaluated using the Alamar blue assay.

For a correct interpretation of the results from the reporter gene assay, the potential of the test materials to reduce cell viability should be assessed. To this end, the Alamar blue assay was used. Fe_3_O_4_-PEG-PLGA nanomaterials were non-cytotoxic towards U2OS cells and only a slight decrease in cell viability (metabolic capacity) was evidenced at the highest tested concentration of 100 μg/ml (Figure 3A). Similarly, TiO_2_ nanomaterials were non-cytotoxic at low concentrations but a markedly decreased viability at the highest concentration of 100 μg/ml (Figure 3B) was noted. In contrast, for Ag nanomaterials, a dose-dependent loss of cell viability was observed (Figure 3C). The potential cytotoxic effects of the reference compounds curcumin, dichlorvos (positive control) and mannitol (negative control) were also evaluated (Figure 4). Neither dichlorvos or mannitol affected cell viability of the U2OS cells, while curcumin at a concentration of 500 nM and higher reduced U2OS cell viability in a dose-dependent manner (Figure 4).

### Nuclear erythroid 2-related factor 2- reporter gene assay

**FIGURE 5:**
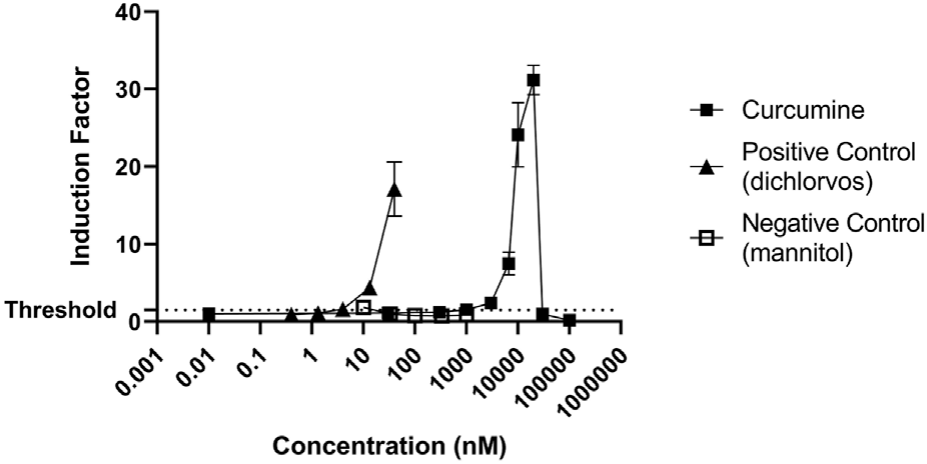
Induction of NRF2 mediated gene expression by the reference compound (curcumin) and negative (mannitol) and positive controls (dichlorvos). The results are presented as induction factor, the fold induction over the solvent control. The data are presented as mean values ± S.D. of three independent experiments.

Next, the induction of the NRF2 pathway was assessed. NRF2-U2OS cells were exposed to increasing concentrations of the reference compound (curcumin), and to the positive and negative controls (Figure 5). Both the reference compound and the positive control (dichlorvos) induced NRF2 mediated gene expression while exposure to mannitol did not (Figure 5).

**FIGURE 6:**
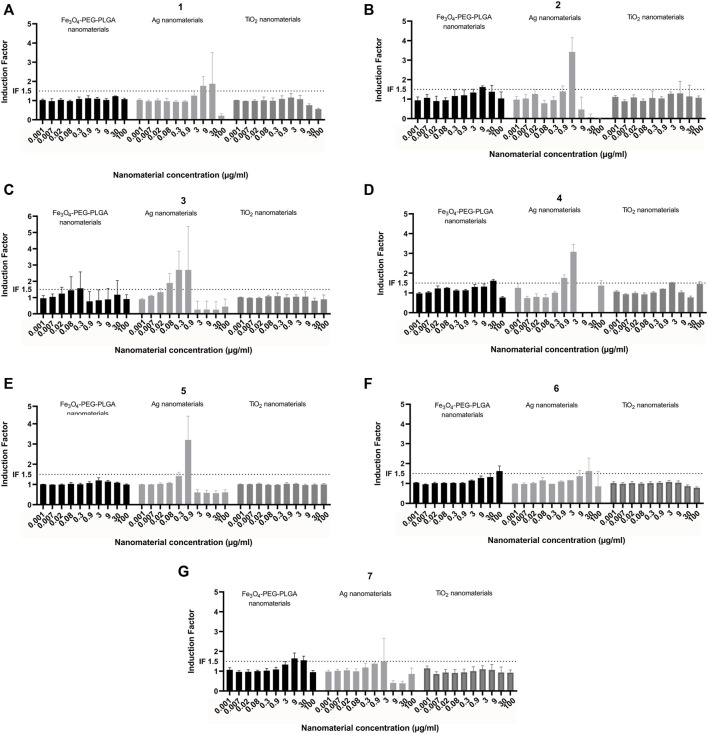
Interlaboratory study. Induction of NRF2 mediated gene expression following exposure of Fe_3_O_4_-PEG-PLGA, Ag nanomaterials and TiO_2_ nanomaterials. Each graph **(A–G)** represents the results of an individual laboratory. Each experiment was performed according to the same harmonized protocol. The results are presented as induction factor, the fold induction over the solvent control. The data are presented as mean values ± S.D. of three independent experiment. The numbers represent the individual participating laboratories. Black bars: Fe_3_O_4_-PEG-PLGA, light grey bars: Ag; dark grey bars: TiO_2_.

Eight laboratories participated in the “round robin” pre-validation study, of which seven used Fe_3_O_4_-PEG-PLGA, Ag, and TiO_2_ nanomaterials (Figure 6), whereas one partner only used Fe_3_O_4_-PEG-PLGA nanomaterials (Supplementary Figure S2). The results consistently showed that TiO_2_ did not induce NRF2-mediated gene expression. However, exposure to Ag nanomaterials induced NRF2-mediated gene expression in a dose-dependent manner in all experiments (Figures 6A–G). Some differences could be observed in the magnitude of responses (i.e., induction factor) of similar concentration in the different laboratories. It was consistently found that the NRF2 mediated gene expression declined at the highest concentrations which likely is due to the cytotoxicity following exposure to the Ag nanomaterials at the higher concentrations. Finally, following exposure to Fe_3_O_4_-PEG-PLGA minimal induction of NRF2-mediated gene expression was observed (Figures 6A–G). Hence, while three of the participating laboratories reported no induction, the results from 5 other laboratories showed a minor induction at 30 or 100 μg/ml, while some reported a lower induction factor for the 100 μg/ml samples compared to 30 μg/ml. Finally the inter and intra-laboratory standard deviations of the assay results were calculated.

**FIGURE 7:**
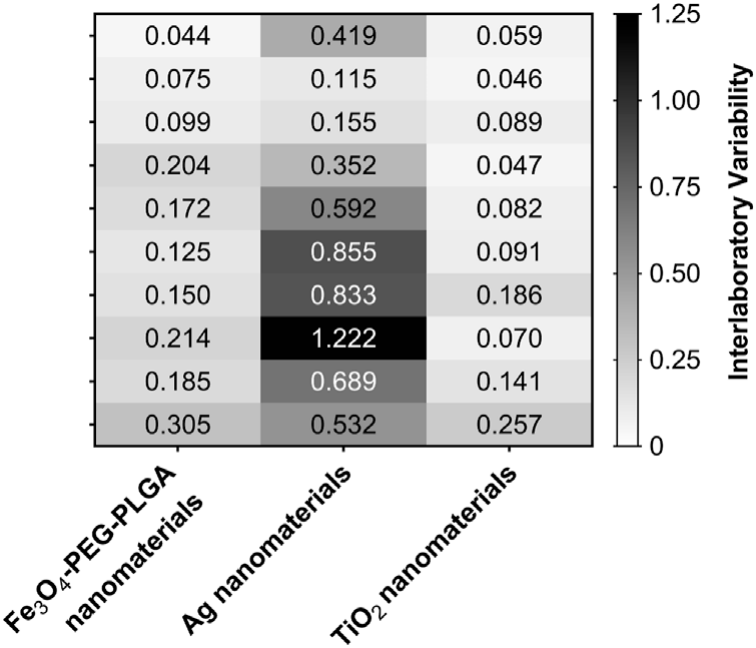
Heatmap of the interlaboratory standard deviation of the NRF2 induction results from all participating laboratories. The combinations of concentration and nanomaterials that resulted in higher variability of assay results across the partner laboratories are indicated by darker boxes in the heatmap.

The inter-laboratory standard deviation ranged from 0.044 to 1.221 with a mean of 0.28 (Figure 7). The mean intra-laboratory standard deviation was 0.16 (Supplementary Figure S3).

To verify the lack of interference of the test materials with the measurement of luciferase activity, the U2OS-NRF2 cells were fixed at the end of exposure by adding 50 µl of paraformaldehyde at 4% in PBS for 30 min at room temperature just before cell lysis to perform the luciferase induction assay as described above. No interference was observed (data not shown).

## Discussion

In this study, we aimed to perform a pre-validation study of a NRF2 reporter gene assay to screen for activation of NRF2 mediated gene expression following exposure to nanobiomaterials, as a proxy for oxidative stress. The pre-validation was conducted through the participation of eight laboratories. Fe_3_O_4_-PEG-PLGA nanomaterials were selected as a representative nanobiomaterial and results were compared to “benchmark” nanomaterials from the JRC namely TiO_2_ (NM101) and Ag (NM300K) along with positive and negative chemical controls. For the TiO_2_ nanomaterials, none of the participating laboratories observed an induction above the threshold. For the Fe_3_O_4_-PEG-PLGA nanomaterials, some laboratories measured an induction just above the threshold while for others the induction levels did not reach the threshold. All laboratories could detect a dose dependent induction following exposure to the Ag nanomaterials (though with different induction factors) indicating that the NRF2 reporter gene assay can be easily applied by different laboratories. Overall, interlaboratory standard deviation was acceptable and the NRF2 reporter gene assay for quantifying oxidative stress caused by nanomaterials is suitable for application in different laboratories. Based on these preliminary findings, we suggest that the assay may be considered for formal validation as an assay for rapid screening of nanobiomaterials.

Engineered nanomaterials can induce ROS production *via* several mechanisms such as the Fenton reaction, redox cycling, and radical generation (Bi and Westerhoff, 2019), which in turn can activate the NRF2 mediated gene expression. Several previous studies have shown that metal oxides including CuO and ZnO nanomaterials can elicit NRF2 activation (Kim et al., 2021; Zhang et al., 2021). Moreover, Ag nanomaterials exposure was found to trigger NRF2 activation which is thus in line with the present findings. The results from the present study showed that Fe_3_O_4_-PEG-PLGA nanomaterials elicited a very modest activation in U2OS-NRF2 cells. However, with respect to Fe_3_O_4_-PEG-PLGA, no literature was found on their potential for the induction of NRF2 mediated gene expression. In a recent study different acellular assays along with a HEK293 cell-based NRF2 reporter assay were compared to study the generation of ROS and antioxidant responses of engineered nanomaterials. It is notable that the HEK293 cell-based NRF2 reporter assay did not show any concentration-dependent reactivity for the Fe-based nanomaterials (Seleci et al., 2022), while Fe_3_O_4_ nanomaterials have been shown to be able to induce oxidative stress in rodents (Wu et al., 2022). The U2OS-NRF2 cells have a low number of receptors expressed, so that the potential for crosstalk between signal transduction pathways is very low. Furthermore, the U2OS cell line has a low overall metabolic capacity so that reactive compounds, or metabolites have a better chance and a higher sensitivity to activate reporter systems. In comparison to the KeratinoSens™ method validated by OECD (Test No. 442D) for *in vitro* skin sensitization which has one ARE-response element upfront of the ARE-reporter construct, the U2OS-NRF2 cells as used in the current study has a tandem of four AREs upstream of the luciferase reporter and may thus be more sensitive to inducers acting *via* the NRF2-pathway. However, care needs to be taken when comparing reporter assays in different cell systems and *in vivo* data as different abundancies of thiol-containing ligands (i.e., GSH and metallothioneins) can influence the presence of intracellular ROS levels (Bobyk et al., 2019) and may thus influence the sensitivity of cell based NRF2 reporter gene assays.

In addition to the aforementioned *in vitro* studies, a number of *in vivo* studies performed in rodents have also shown that nanomaterials can trigger NRF2 activation. For instance, Sun et al. (2012) showed that long-term exposure to TiO_2_ nanomaterials induced the expression of NRF2, heme oxygenase 1 (HO-1), and glutamate-cysteine ligase catalytic subunit (GCLC). Other investigators have reported that intratracheal administration of ZnO nanomaterials induced elevation of NRF2 and HO-1 expression in the aorta of mice (Zhang et al., 2021). Furthermore, members of the BIORIMA consortium previously investigated the role of NRF2 in pulmonary inflammation following exposure to ZnO nanomaterials using *Nrf2*-null mice (Sehsah et al., 2019). Mice were administered 20 nm ZnO nanomaterials via pharyngeal aspiration and the study demonstrated infiltration of inflammatory cells in the lung of mice, but minimally induced NRF2-dependent antioxidant enzymes. The authors concluded that NRF2 plays a role in negative regulation on ZnO nanoparticle-induced neutrophil migration (Sehsah et al., 2019).

Several studies have been undertaken in recent years to improve the quality of nanotoxicological investigations including a number of interlaboratory comparisons (aka round robins). For instance, a US consortium funded by the NIEHS conducted cell-based assays on a panel of nanomaterials including several forms of TiO_2_ and ZnO nanomaterials as well as multi-walled carbon nanotubes focusing on cell viability and cytokine (IL-1β) production (Xia et al., 2013). The importance of using well-characterized nanomaterials and positive and negative controls was emphasized. Several pan-European projects have also addressed the harmonization of *in vitro* test protocols for the assessment of nanomaterials (e.g., Dusinska et al., 2015; Farcal et al., 2015; Kermanizadeh et al., 2016; Piret et al., 2017). These efforts have put a spotlight on the crucial importance of harmonized test protocols while acknowledging that the path to regulatory-relevant results can be both arduous and long (Teunenbroek et al., 2017).

The OECD Working Party on Manufactured Nanomaterials (WPMN) has reviewed the need for adaptation of the existing OECD Test Guidelines (TGs) and Guidance Documents (GDs) as well as developing new TGs and GDs to address nanomaterials (Rasmussen et al., 2019). Indeed, in the frame of the so-called Malta Initiative, 18 European countries, several Directorates-General of the European Commission, the European Chemicals Agency (ECHA), and other organizations collaborate with the aim of making legislation enforceable, in particular for chemicals (Mech et al., 2022). This European action is currently focused on amending the OECD TGs with respect to nanomaterials to ensure that they are fit-for-purpose. The present reporter gene assay which reflects an important biological endpoint namely oxidative stress is well aligned with these efforts, although further validation is certainly required.

## Conclusion

In conclusion, we have successfully performed a pre-validation “round robin” using the NRF2 reporter gene assay using Fe_3_O_4_-PEG-PLGA vs. TiO_2_ (NM101) and Ag (NM300K) nanomaterials. The assay was readily adopted by different laboratories. It is worth noting that other reporter gene assays have previously been subjected to validation and that the estrogen receptor (ER) reporter gene assay and androgen receptor (AR)-reporter gene have recently been included in OECD TG 455 and TG 458, respectively. We suggest that the results of the present interlaboratory study may serve as a starting point for a larger validation study to develop the NRF2 gene reporter assay for the screening of the induction of oxidative stress responses triggered by nanobiomaterials. Indeed, high-throughput screening using *in vitro* assays could speed up the hazard assessment of nano (bio) materials (Fadeel et al., 2018).

## Data Availability

The raw data supporting the conclusions of this article will be made available by the authors, without undue reservation.
