# Supplementary material for: Pre-validation of a reporter gene assay for oxidative stress for the rapid screening of nanobiomaterials
Source: Front Toxicol. 2022 Sep 5;4:974429. doi: 10.3389/ftox.2022.974429 (PMC9511406; doi:10.3389/ftox.2022.974429)
Supplement: Supplementary file 1 [file DataSheet1.docx]

Supplementary Information

**Pre-validation of a reporter gene assay for oxidative stress for the rapid screening of nanobiomaterials**

*Sebastin Martin^1^, Laura de Haan^2^, Ignacio Miro Estruch^2^, Kai Moritz Eder^3^, Anne Marzi^3^, Jürgen Schnekenburger^3^, Magda Blosi^4^, Anna Costa^4^, Giulia Antonello^5^, Enrico Bergamaschi^5^, Chiara Riganti^5^, David Beal^6^, Marie Carrière^6^, Olivier Taché^7^, Gary Hutchison^8^, Eva Malone^8^, Lesley Young^8^, Luisa Campagnolo^9^, Fabio La Civita^9^,* ***Antonio Pietroiusti^9,#^****, Stéphanie Devineau^10^, Armelle Baeza^10^, Sonja Boland^10^, Cai Zong^11^, Gaku Ichihara^11^, Bengt Fadeel^1^, and Hans Bouwmeester^2,^**

^1^Division of Molecular Toxicology, Institute of Environmental Medicine, Karolinska Institutet, Stockholm, Sweden,

^2^Division of Toxicology, Wageningen University and Research, Wageningen, The Netherlands,

^3^Biomedical Technology Center, Westfälische Wilhelms-University, Münster, Germany,

^4^Institute of Science and Technology for Ceramics (ISTEC), CNR, Faenza, Italy,

^5^Department of Public Health and Pediatrics, University of Torino, Torino, Italy,

^6^Université Grenoble-Alpes, CEA, CNRS, IRIG, SyMMES, Grenoble, France,

^7^Université Paris-Saclay, CEA, CNRS, NIMBE, Gif-sur-Yvette, France,

^8^School of Applied Sciences, Edinburgh Napier University, Edinburgh, United Kingdom,

^9^Department of Biomedicine and Prevention, University of Rome Tor Vergata, Rome, Italy,

^10^ Université Paris Cité, CNRS, Unité de Biologie Fonctionnelle et Adaptative, F-75013 Paris, France,

^11^Department of Occupational and Environmental Health, Tokyo University of Science, Tokyo, Japan

*Correspondence. E-mail: [hans.bouwmeester@wur.nl](mailto:hans.bouwmeester@wur.nl)

## ***^#^***Present address: Saint Camillus International University of Health Sciences, Rome, Italy.


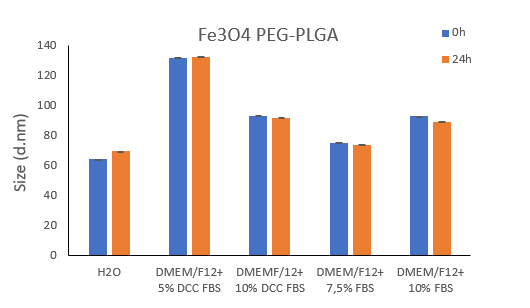

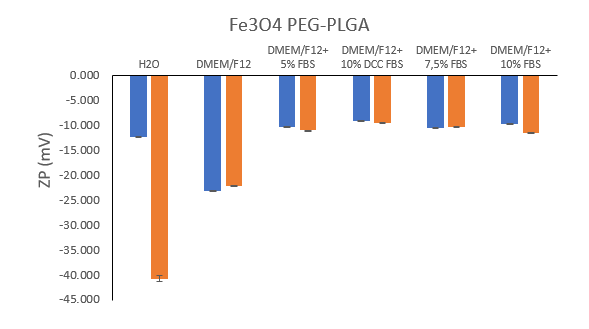


**B**

**A**

Figure S1. Characterization of Fe_3_O_4_-PEG-PLGA. (a) Hydrodynamic size and (b) zeta potential of Fe_3_O_4_-PEG-PLGA in relevant biological media. DMEM/F12, Dulbecco’s Modified Eagle Medium with Ham’s Nutrient Mixture F-12; FBS, fetal bovine serum; DCC-FCS, dextran-coated charcoal-stripped fetal calf serum.

**
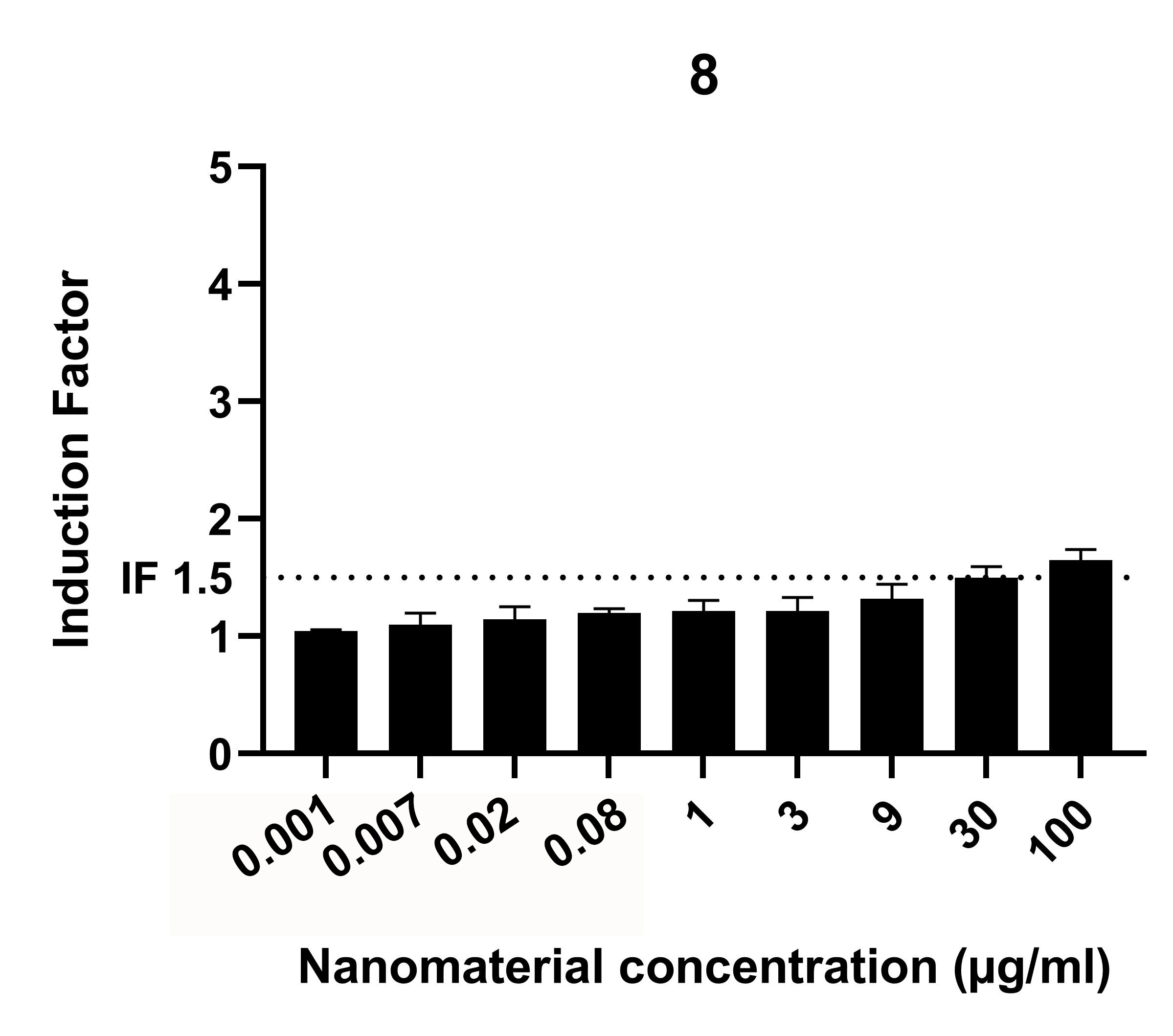
**

Figure S2. NRF2 mediated gene expression in response to Fe_3_O_4_-PEG-PLGA.

The data are from participating laboratory 8 (no data on TiO_2_ and Ag nanomaterials (i.e., NM101 and NM300K respectively).


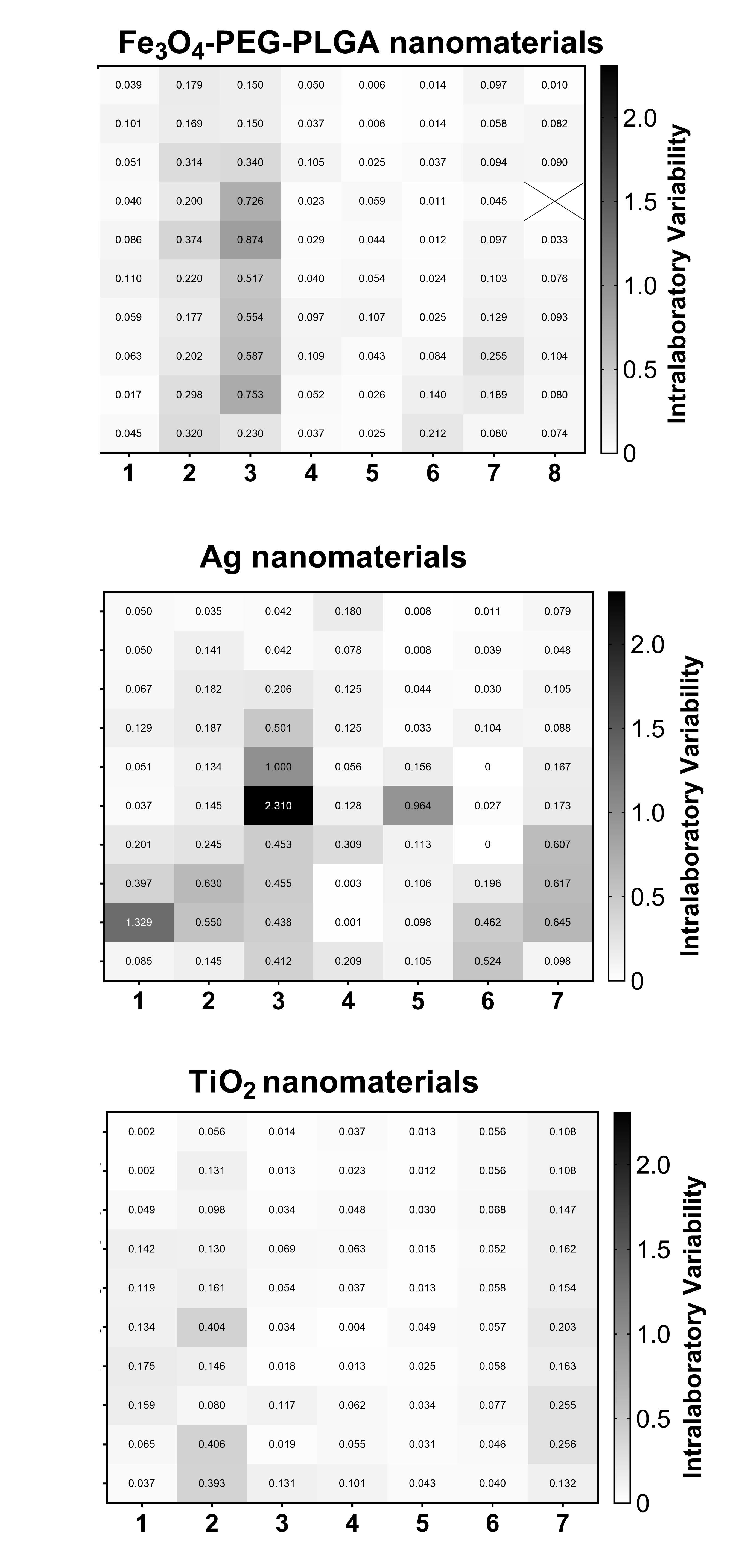


Figure S3. Heatmaps of the intra-laboratory standard deviations of all NRF2 induction results performed with the U2OS cell line. The combinations of concentration and nanomaterials that resulted in higher variability of assay results are indicated by darker boxes in the greyscale heatmap. The numbers represent the individual participating laboratories (1 – 8) (Fe_3_O_4_-PEG-PLGA) and (1 – 7) (NM300 and NM101).
